# Calcium Sensing Receptor Modulates Extracellular Calcium Entry and Proliferation via TRPC3/6 Channels in Cultured Human Mesangial Cells

**DOI:** 10.1371/journal.pone.0098777

**Published:** 2014-06-06

**Authors:** Kexin Meng, Jia Xu, Chengwei Zhang, Rui Zhang, He Yang, Chang Liao, Jundong Jiao

**Affiliations:** 1 Department of Nephrology, The Second Affiliated Hospital, Harbin Medical University, Harbin, China; 2 Institute of Nephrology, Harbin Medical University, Harbin, China; 3 Department of Nephrology, The Fourth Affiliated Hospital, Harbin Medical University, Harbin, China; Fondazione IRCCS Ospedale Maggiore Policlinico & Fondazione D'Amico per la Ricerca sulle Malattie Renali, Italy

## Abstract

Calcium-sensing receptor (CaSR) has been demonstrated to be present in several tissues and cells unrelated to systemic calcium homeostasis, where it regulates a series of diverse cellular functions. A previous study indicated that CaSR is expressed in mouse glomerular mesangial cells (MCs), and stimulation of CaSR induces cell proliferation. However, the signaling cascades initiated by CaSR activation in MCs are currently unknown. In this study, our data demonstrate that CaSR mRNA and protein are expressed in a human mesangial cell line. Activating CaSR with high extracellular Ca^2+^ concentration ([Ca^2+^]_o_) or spermine induces a phospholipase C (PLC)-dependent increase in intracellular Ca^2+^ concentration ([Ca^2+^]_i_). Interestingly, the CaSR activation-induced increase in [Ca^2+^]_i_ results not only from intracellular Ca^2+^ release from internal stores but also from canonical transient receptor potential (TRPC)-dependent Ca^2+^ influx. This increase in Ca^2+^ was attenuated by treatment with a nonselective TRPC channel blocker but not by treatment with a voltage-gated calcium blocker or Na^+^/Ca^2+^ exchanger inhibitor. Furthermore, stimulation of CaSR by high [Ca^2+^]_o_ enhanced the expression of TRPC3 and TRPC6 but not TRPC1 and TRPC4, and siRNA targeting TRPC3 and TRPC6 attenuated the CaSR activation-induced [Ca^2+^]_i_ increase. Further experiments indicate that 1-oleoyl-2-acetyl-sn-glycerol (OAG), a known activator of receptor-operated calcium channels, significantly enhances the CaSR activation-induced [Ca^2+^]_i_ increase. Moreover, under conditions in which intracellular stores were already depleted with thapsigargin (TG), CaSR agonists also induced an increase in [Ca^2+^]_i_, suggesting that calcium influx stimulated by CaSR agonists does not require the release of calcium stores. Finally, our data indicate that pharmacological inhibition and knock down of TRPC3 and TRPC6 attenuates the CaSR activation-induced cell proliferation in human MCs. With these data, we conclude that CaSR activation mediates Ca^2+^ influx and cell proliferation via TRPC3 and TRPC6 in human MCs.

## Introduction

Calcium-sensing receptor (CaSR), a cell-surface protein, is highly expressed in tissues and cells involved in systemic calcium homeostasis, including the parathyroid gland, kidney, and bone, where it contributes to the maintenance of systemic calcium within a narrow physiological window [1]. However, CaSR is also expressed in many other tissues and cells that are not primarily involved in extracellular calcium homeostasis, such as in the brain, skin, lungs, suggesting that this receptor plays additional physiological roles in the regulation of cell functions, such as cellular proliferation [2], differentiation [3] and apoptosis [4]. In the kidney, CaSR is well known to regulate calcium excretion and absorption in the renal tubules [5]. Interestingly, recent evidence indicates that CaSR is also expressed in glomeruli, and pharmacological activation of CaSR by the calcimimetic R-568 exerts a direct nephroprotective effect at the glomerular podocyte level [6], [7]. A previous study showed that CaSR was expressed in mouse glomerular mesangial cells (MCs), and stimulation of CaSR induced cell proliferation [8]. However, nothing is currently known about the signaling cascades initiated by CaSR activation in MCs.

Although downstream effects can be highly varied, the first reactions following CaSR activation are common; stimulation of CaSR evokes an increase in intracellular Ca^2+^ concentration ([Ca^2+^]_i_) [9]. CaSR belongs to family C of the G protein-coupled receptor superfamily. Stimulation of CaSR by an increase in extracellular Ca^2+^ concentration ([Ca^2+^]_o_) or a polyamine (such as spermine) activates phospholipase C (PLC), which converts phosphatidylinositol 4,5-bisphosphate into inositol-1,4,5-trisphosphate (IP_3_) and diacylglycerol (DAG). IP_3_ triggers Ca^2+^ release from internal stores, resulting in an increase in [Ca^2+^]_i_. However, the concomitant store depletion might mediate store-operated calcium entry (SOCE) through store-operated channels (SOCs) in the plasma membrane. Moreover, DAG can cause receptor-operated calcium entry (ROCE) by activating receptor-operated channels (ROCs). IP_3_-mediated Ca^2+^ release, SOCE and ROCE all likely contribute to the increase in [Ca^2+^]_i_ upon activation of CaSR. IP_3_-mediated Ca^2+^ release in response to CaSR stimulation has been widely investigated in many cell types; however, relatively little is known about calcium entry mechanism upon CaSR activation. SOCs and, in many cases ROCs, have been identified as canonical transient receptor potential (TRPC) channels. Furthermore, several studies indicated that TRPC channels are involved in the CaSR stimulation-induced calcium influx in some cell types, such as salivary ductal cells [10], MCF-7 breast cancer cells [2], aortic smooth muscle cells [11], keratinocytes [12], pulmonary neuroendocrine cells [13] and osteoclasts [14].

Studies from our laboratory and other laboratories have demonstrated that human MCs express TRPC channel proteins, including isoforms of TRPC1, 3, 4, and 6 [15], [16]. In the present study, we investigated the role of TRPC channels in the CaSR activation-induced calcium influx and subsequent cell proliferation in human MCs. We determined that CaSR activation mediated TRPC3- and TRPC6-dependent calcium entry in a store-independent manner. Furthermore, knockdown or pharmacological blockage of TRPC3 and TRPC6 inhibited the CaSR agonist-induced cell proliferation.

## Materials and Methods

### Cell culture and transfection

An stable human mesangial cell line (kindly donated by Dr. J. D. Sraer, Hopital Tenon, Paris, France) was established by transfection and immortalization by the viral oncogene large T-SV40 of human mesangial cells isolated from normal human glomeruli [17], and were cultured as described previously [16]. Briefly, the cells were cultured in RPMI1640 medium (HyClone, USA) containing 1 mM Ca^2+^ supplemented with 10% fetal bovine serum (HyClone, USA) in 5% CO_2_ at 37°C. Human MCs between passages 3 and 15 were used. A human breast cancer cell line MCF-7 was obtained from the Cell Bank of the Chinese Academy of Science (Shanghai, China), maintained in 5% CO_2_ at 37°C in DMEM medium (HyClone, USA) containing 10% fetal bovine serum (HyClone, USA). Human MCs were transiently transfected with human TRPC3 siRNA, TRPC6 siRNA or scrambled siRNA (Santa Cruz, USA) using the Xtreme GENE siRNA transfection reagent (Roche, Germany) according to the manufacturer's instructions. The transfected cells were assayed 24 to 48 h post-transfection.

### Reverse transcription PCR and quantitative real-time PCR

Reverse transcription was performed using an RT system (Eppendorf Mastercycler, Hamburg, Germany) and a 10 µl reaction mixture. A High Capacity cDNA RT Kit (ABI Applied Biosystems, USA) was used for the initiation of cDNA synthesis. The primer sequences used to amplify CaSR, TRPC1, TRPC3, TRPC4 and TRPC6 were as follows (5′-3′):

CaSR sense CGGGGTACCTTAAGCACCTACGGCATCTAA,

and antisense GCTCTAGAGTTAACGCGATCCCAAAGGGCTC;

TRPC1 sense CGCCGAACGAGGTGAT,

and antisense GCACGCCAGCAAGAAA;

TRPC3 sense CGGCAACATCCCAGTG,

and antisense CGTAGAAGTCGTCGTCCTG;

TRPC4 sense CTCTGGTTGTTCTACTCAACATG,

and antisense CCTGTTGACGAGCAACTTCTTCT;

TRPC6 sense GCCAATGAGCATCTGGAAAT,

and antisense TGGAGTCACATCATGGGAGA.

PCR cycling conditions for CaSR included one cycle of 10 min at 95°C, 35 cycles of 30 s at 95°C, 30 s at 55°C and 60 s at 72°C, and one cycle of 10 min at 72°C. The PCR products of CaSR were then separated on a 1% agarose gel and stained with ethidium bromide. Reverse transcriptase was omitted as a negative control for the RT-PCR to eliminate amplification from contaminating genomic DNA. All real-time PCR experiments were performed with SYBR Green PCR MasterMix (ABI Applied Biosystems, UK) using an ABI PRISM 7500 (ABI Applied Biosystems, USA). GAPDH was used as the internal control, and ΔΔCt was calculated for each sample with the expression levels indicated by values of 2^−ΔΔCt^.

### Western blot

Western blot was performed using a standard protocol. Human MCs were starved for 24 h in a serum-free medium prior to stimulation with high [Ca^2+^]_o_. At the end of the 24 h incubation, the cells were harvested for western blot analysis. Anti-CaSR antibody (Affinity BioReagents, USA), anti-TRPC1, -TRPC3, -TRPC4, or -TRPC6 antibodies (Alomone Labs, Israel) or an anti-actin antibody (Santa Cruz, USA) were used as primary antibodies. Fluorescence-conjugated goat anti-rabbit or goat anti-mouse IgG antibodies (Invitrogen, USA) were used as secondary antibodies. Western blot bands were quantified using the Odyssey infrared imaging system (LI-COR Bioscience, USA).

### Immunofluorescence

Immunofluorescence staining was performed on cultured MCs growing on coverslips using a standard protocol. Briefly, cells were fixed with 4% paraformaldehyde for 15 min and permeabilized with 0.4% Triton X-100 in PBS for 60 min at room temperature. The nonspecific binding sites were blocked with 50% goat serum in PBS for 60 min at 37°C. The cells were then incubated overnight at 4°C with anti-CaSR antibody (Affinity BioReagents, USA). After washing, cells were stained with Alexa Fluor 594 conjugated to goat anti-mouse secondary antibody (Molecular Probes, Eugene, OR) for 60 min at room temperature. Secondary antibody without prior antibody treatment was also included as negative controls. Cells were then stained with DAPI (Sigma, USA) for 15 min at room temperature to detect nuclei. After washing, samples were examined under a laser scanning confocal microscope (FV300; Olympus, Japan). Calibrations were performed immediately following each experiment. More than 50 cells were inspected per experiment, and photos of cells with typical morphology and staining are presented.

### Fluorescence measurement of [Ca^2+^]_i_

MCs were grown on coverslips and loaded in 1% physiological saline solution containing Pluronic F-127 (0.03%, Sigma, USA) and Fluo-3/AM (3 µM, Molecular Probes, USA) at 37°C for 45 min. After washing, the coverslips with cells were placed in a chamber containing HEPES-buffered Na^+^ medium (HBM) that consists of the following (in mM): 137 NaCl, 5 KCl, 1 CaCl_2_, 1.2 MgCl_2,_ 0.44 KH_2_PO4, 4.2 NaHCO_3_, 10 glucose, and 20 HEPES; the pH was adjusted to 7.4 with NaOH. For the Ca^2+^-free HBM, Ca^2+^ was omitted. MCs were then stimulated with a variety of agonists or inhibitors as described in the results, including spermine, NPS2390, U73122, thapsigargin (TG), SKF96365, 2-aminoethoxydiphenyl borate (2APB), efonidipine, 1-oleoyl-2-acetyl-sn-glycerol (OAG) (all from Sigma Chemical Co., USA), and SN-6 (Tocris Bioscience, Bristol, UK). The fluorescence intensity of Fluo-3 in the cells was recorded by a laserconfocal scanning microscope (FV300; Olympus, Japan). The [Ca^2+^]_i_ was expressed as a pseudo-ratio value of the actual fluorescence intensity divided by the average baseline fluorescence intensity. Calibrations were performed immediately following each experiment. Data from 20 to 40 cells were summarized in a single run, and at least three independent experiments were conducted.

### Cell proliferation assay

Cell proliferation was measured by a Cell Proliferation ELISA BrdU kit (Roche, Germany) according to the manufacturer's protocol. Cells were seeded in a 96-well plate (5000 cells/well) and cultured for 24 h. After starvation for another 24 h in serum-free medium, cells were then incubated in the same medium supplemented with different [Ca^2+^]_o_ in the presence or absence of various inhibitors for 24 h. Eight hours before the end of incubation, BrdU was added to the medium, and cells then were continually incubated for 8 hours. The absorbance at 450 nm (reference wavelength 630 nm) was measured with a scanning multi-well spectrophotometer (Amersham Pharmacia Biotech). The absorbance values correlate directly to the amount of DNA synthesis and therefore to the number of proliferating cells in culture. Stimulation is expressed as fold proliferation over basal growth of the control set as unity.

### Statistical analysis

Data are presented as the means ± SEMs with the indicated number (n) of experiments. Statistical analyses were performed using an unpaired t-test (SPSS 16.0), and graphs were plotted in GraphPad Prism 5 (GraphPad Software, Inc.). P<0.05 was considered statistically significant.

## Results

### CaSR is expressed in human MCs

**Figure 1:**
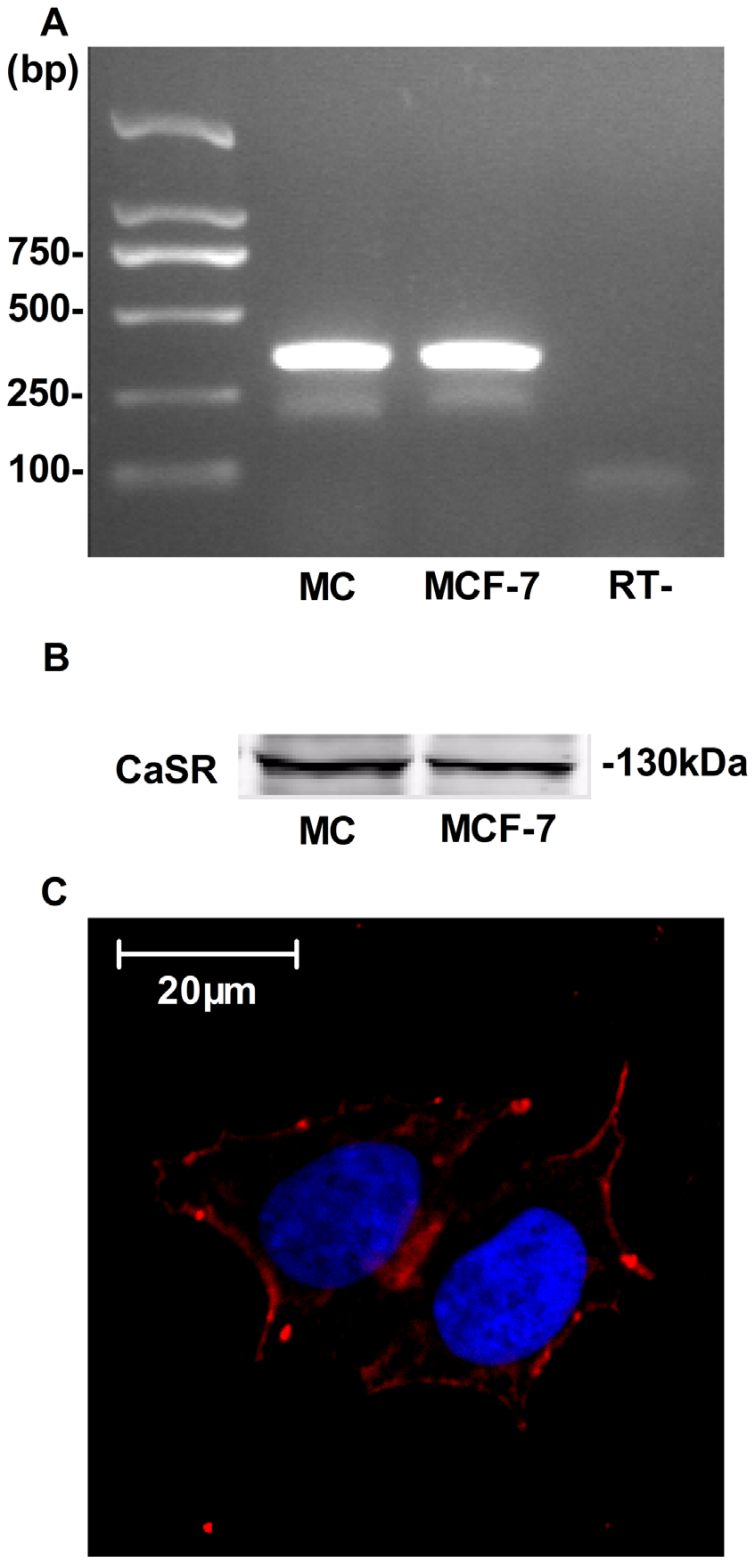
Expression of CaSR mRNA and protein in human MCs. (A) RT-PCR was performed on total RNA extracted from human MCs or MCF-7 cells (a human breast cancer cell line used as a positive control) using primers specific for the human CaSR to detect CaSR mRNA expression. A PCR product of the expected size (424 bp) was observed. In the absence of reverse transcriptase (RT-), no PCR-amplified products were detected. (B) Western blot analysis was performed to detect the protein expression of CaSR in human MCs. A 130 kDa band was found in both human MCs and in the positive control of MCF-7 cells. (C) Immunofluorescence detection of CaSR localization in human MCs with anti-CaSR antibody. Red color indicates labeling with CaSR, and blue color shows DAPI-stained nuclei. Scale bar, 20 µm. At least 50 cells were examined, and the present images represent typical staining pattern for the majority of examined cells.

To determine whether CaSR mRNA is expressed in cultured human MCs, RT-PCR was performed using specific primers for CaSR. As shown in Fig. 1A, a PCR product of the expected size (424 bp) was observed. In the absence of reverse transcriptase, no PCR-amplified products were detected, indicating that the tested RNA samples were free of genomic contamination. As a positive control, an RT-PCR product from MCF-7 cells revealed a band of the same size as the human MCs. The expression of CaSR protein in human MCs was explored by Western blot analysis and immunostaining. As shown in Fig. 1B, a 130 kDa band, corresponding with the mature CaSR, was found in both human MCs and in the positive control of MCF-7 cells. Immunostaining showed that CaSR protein was mainly localized at the plasma membrane along with some cytoplasmic localization (Fig. 1C). Taken together, these data demonstrate that CaSR is present in cultured human MCs.

### Activation of CaSR stimulates an increase [Ca^2+^]_i_ in human MCs

**Figure 2:**
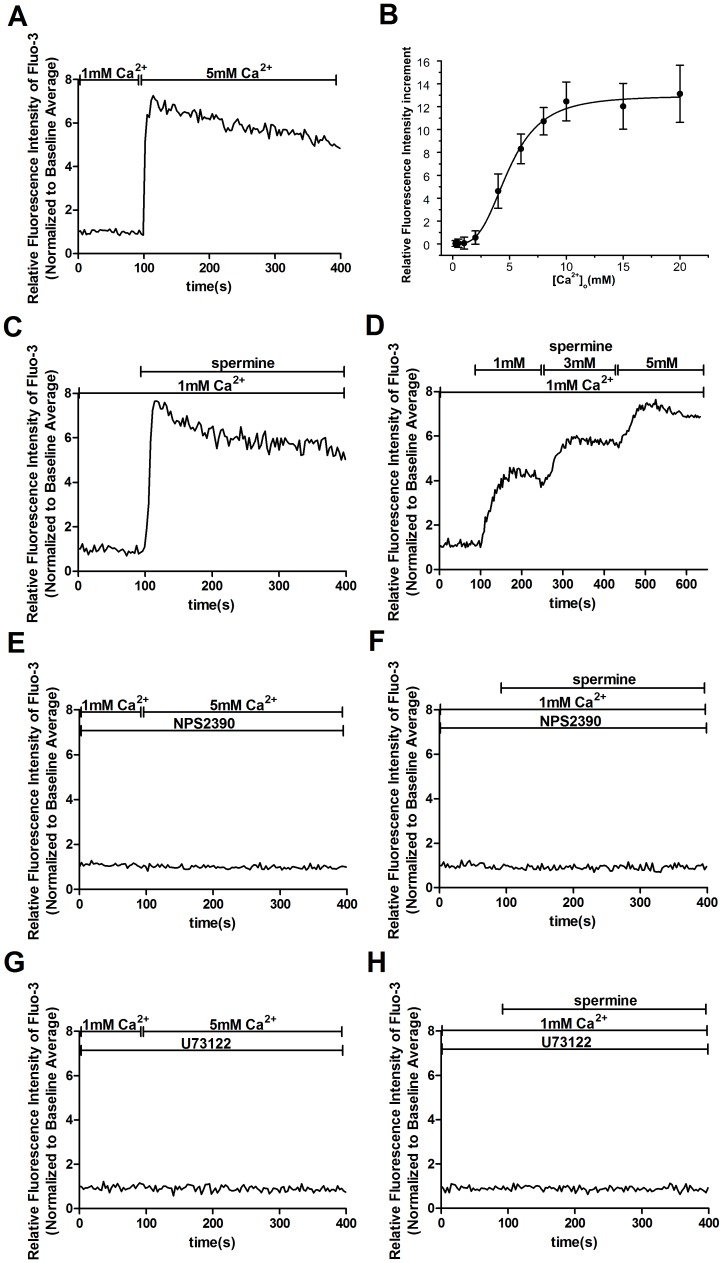
Activation of CaSR induces an increase in [Ca^2+^]_i_ in human MCs. [Ca^2+^]_i_ dynamics were monitored by Fura-3 fluorescence methods with a laserconfocal scanning microscope. Representative traces are shown in A and C-H. (A) An increase in [Ca^2+^]_o_ from 1 to 5 mM evokes a rapid peak and a subsequent sustained elevated level of [Ca^2+^]_i_. (B) Summary of data showing a concentration-dependent relationship for the effect of [Ca^2+^]_o_ stimulation or peak [Ca^2+^]_i_ responses. Human MCs were stimulated with different concentrations of [Ca^2+^]_o_ (0.5–20 mM). Data are shown as the means ± SEs of 40–50 cells. (C) In the presence of extracellular Ca^2+^ (1 mM), addition of 3 mM spermine evokes a rapid peak of [Ca^2+^]_i_ and a subsequent sustained increase in [Ca^2+^]_i_. (D) Dose-dependence of the spermine-induced increase in [Ca^2+^]_i_. Cells were stimulated by different concentrations of spermine (1–5 mM) in medium containing 1 mM Ca^2+^. (E, F) Pretreatment with 10 µM NPS2390 for 20 min inhibits the 5 mM [Ca^2+^]_o_(E)- or 3 mM spermine(F)-induced [Ca^2+^]_i_ increase (p<0.01 vs. NPS2390(-), n = 6), respectively. (G, H) Treatment with 5 mM [Ca^2+^]_o_(G) or 3 mM spermine(H) induced an increase in [Ca^2+^]_i_, and this increase is inhibited by pretreatment with 30 µM U73122 for 20 min (p<0.01 vs. U73122(-), n = 6), respectively. The results were from at least three independent experiments, and each experiment measured 20 to 40 cells.

To evaluate if the expression of CaSR protein is associated with the presence of functional receptors, Fluo-3/AM-loaded human MCs were stimulated by known CaSR agonists. As shown in Fig. 2A, a change in [Ca^2+^]_o_ from 1 to 5 mM evoked a rapid peak of [Ca^2+^]_i_ and a subsequent sustained increase in [Ca^2+^]_i_. The increase in the [Ca^2+^]_i_ due to the [Ca^2+^]_o_ occurred in a concentration-dependent manner, as shown in Fig. 2B. The half maximal effective concentration (EC50) of [Ca^2+^]_o_ that was necessary to achieve the [Ca^2+^]_i_ response in the human MCs was approximately 4.93 mM. A similar effect was observed with the use of 3 mM spermine, another CaSR agonist (Fig. 2C), indicating that the observed effect of CaSR activation was not agonist-specific. Additionally, the increase in [Ca^2+^]_i_ induced by spermine was dose-dependent (Fig. 2D). Both the 5 mM [Ca^2+^]_o_- and 3 mM spermine-induced [Ca^2+^]_i_ increases were significantly inhibited by pretreatment with 10 µM NPS2390, an antagonist of CaSR (Fig. 2E and 2F). To evaluate whether the increase in [Ca^2+^]_i_ induced by CaSR activation involves a PLC-dependent pathway, cells were stimulated with CaSR agonists in the presence of the PLC inhibitor U73122. The [Ca^2+^]_i_ increase induced by 5 mM [Ca^2+^]_o_ or 3 mM spermine was significantly inhibited by pretreatment with 30 µM U73122, as shown in Fig. 2G and 2H, respectively. Taken together, these data confirm that CaSR protein is functionally expressed in human MC and activates a PLC-dependent [Ca^2+^]_i_ increase.

### CaSR activation induces both intracellular Ca^2+^ release and TRPC-dependent Ca^2+^ influx

**Figure 3:**
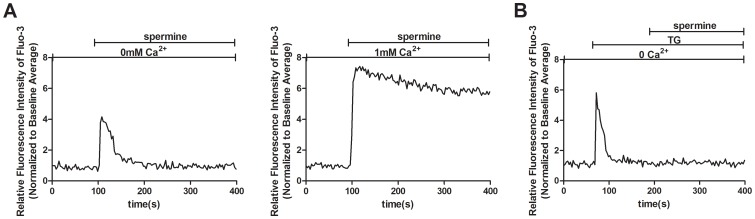
CaSR activation induces intracellular Ca^2+^ release. [Ca^2+^]_i_ dynamics were monitored by Fura-3 fluorescence methods. (A) Representative traces showing that 3 mM spermine induces an increase in [Ca^2+^]_i_ even in the absence of extracellular Ca^2+^(left). Cells were bathed in Ca^2+^-free solution followed by addition of 3 mM spermine. In the presence of extracellular 1 mM Ca^2+^, addition of 3 mM spermine evokes a rapid peak of [Ca^2+^]_i_ and a subsequent sustained increase in [Ca^2+^]_i_ (right). (B) Depletion of internal Ca^2+^ stores by 1 µM thapsigargin (TG) abolishes the 3 mM spermine-induced [Ca^2+^]_i_ signaling in the absence of extracellular Ca^2+^. Cells bathed in Ca^2+^-free solution were stimulated with 1 µM TG, and then, 3 mM spermine was added to the bath solution. The results were from at least three independent experiments, and each experiment measured 20 to 40 cells.

Because Ca^2+^ mobilization from intracellular stores by CaSR agonists has been shown in many cell types, we investigated whether similar effects of CaSR agonists occur in human MCs. Cells were stimulated by spermine in the absence of extracellular Ca^2+^. As shown in Fig. 3A, 3 mM spermine induced an increase in [Ca^2+^]_i_ in Ca^2+^-free solutions. Accordingly, no Ca^2+^ signal was ever observed after store depletion by 1 µM thapsigargin (TG), an endoplasmic reticulum Ca^2+^-ATPase inhibitor, further indicating that CaSR agonists stimulate Ca^2+^ release from intracellular stores (Fig. 3B). Interestingly, as shown in Fig. 3A, the spermine-induced [Ca^2+^]_i_ increase in the absence of extracellular Ca^2+^ was smaller than that observed in the presence of 1 mM [Ca^2+^]_o_ and had no subsequent sustained increase in [Ca^2+^]_i_, suggesting that extracellular Ca^2+^ influx is most likely involved in the increase of [Ca^2+^]_i_ by CaSR agonists in human MCs.

**Figure 4:**
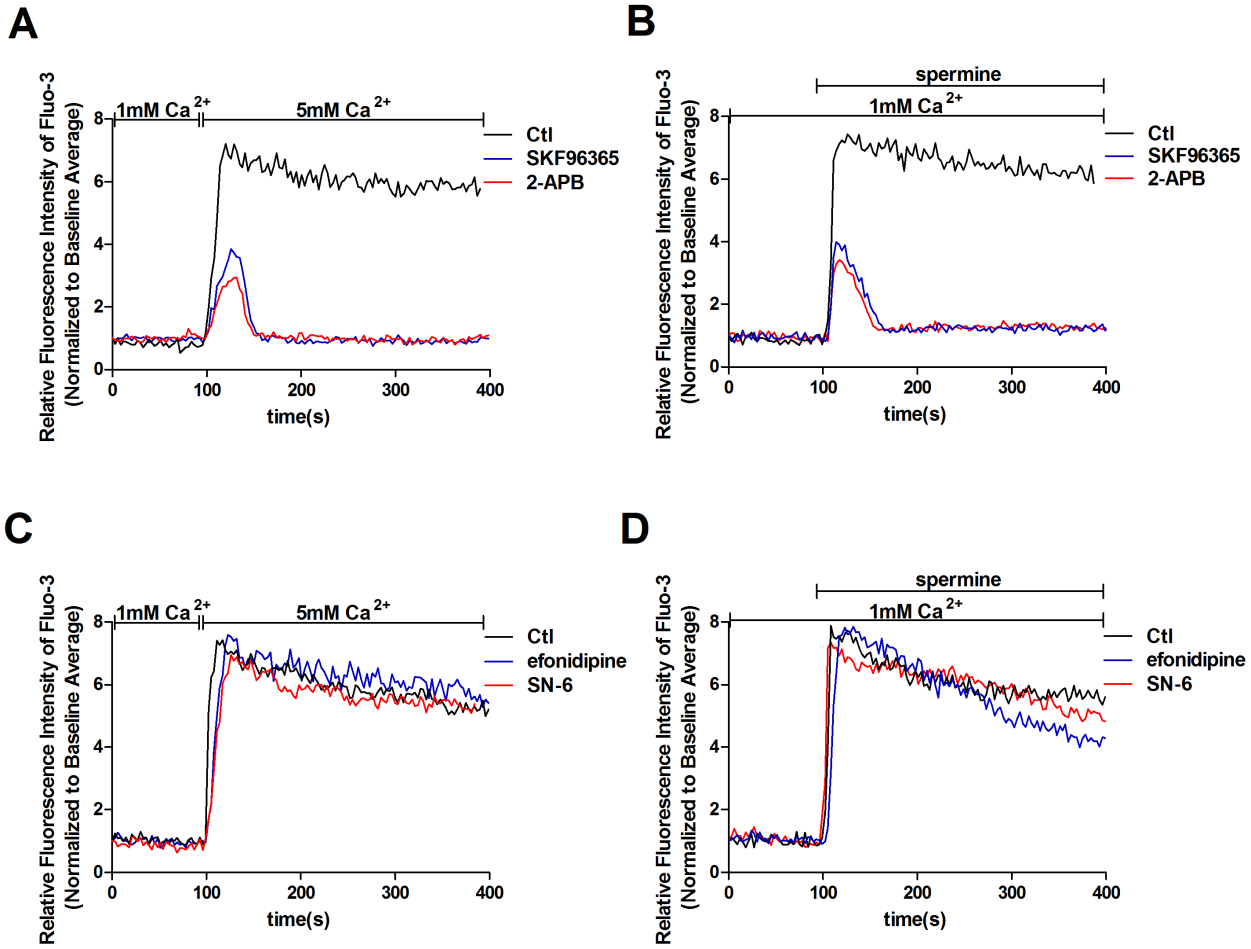
CaSR activation induces TRPC-dependent Ca^2+^ signaling. [Ca^2+^]_i_ dynamics were monitored by Fura-3 fluorescence methods. Representative traces are shown in A–D. (A) The 5 mM [Ca^2+^]_o_-induced [Ca^2+^]_i_ increase is inhibited in the presence of TRPC channel blockers (p<0.05 vs. Ctl, n = 5). Cells were pretreated with 50 µM SKF96365 or 100 µM 2-APB for 20 min in medium containing 1 mM Ca^2+^ followed by a change in [Ca^2+^]_o_ from 1 to 5 mM. (B) In the presence of TRPC channel blockers, the 3 mM spermine-induced [Ca^2+^]_i_ increase is inhibited (p<0.05 vs. Ctl, n = 5). Cells were pretreated with 50 µM SKF96365 or 100 µM 2-APB for 20 min in medium containing 1 mM Ca^2+^ followed by addition of 3 mM spermine. (C) Pretreatment of the cells with efonidipine (10 µM) or SN-6 (10 µM) for 20 min had no apparent effect on the 5 mM [Ca^2+^]_o_-induced [Ca^2+^]_i_ signaling (p>0.05, n = 5). (D) Pretreatment of the cells with efonidipine (10 µM) or SN-6 (10 µM) for 20 min had no apparent effect on the 3 mM spermine-induced [Ca^2+^]_i_ increase (p>0.05, n = 5). All other additions are indicated in the figure. The results were from at least three independent experiments, and each experiment measured 20 to 40 cells.

Because TPRC channels, voltage-gated calcium channels and Na^+^/Ca^2+^ exchangers are the main pathways for Ca^2+^ influx in MCs [18], we examined the role of these pathways in CaSR agonist-induced [Ca^2+^]_i_ increase. As shown in Fig. 4A and 4B, pretreatment with SKF96365 (50 µM) or 2-APB (100 µM), nonselective TRPC channel blockers [19], significantly inhibited the 5 mM [Ca^2+^]_o_- and 3 mM spermine-induced [Ca^2+^]_i_ increase, whereas pretreatment with efonidipine (10 µM, a voltage-gated calcium blocker) or SN-6 (10 µM, an specific inhibitor of NCX) had no apparent effect (Fig. 4C and 4D). These data indicate that TRPC-dependent Ca^2+^ entry is involved in the CaSR agonist-induced [Ca^2+^]_i_ increase. Therefore, in the following experiments, we focus on TRPC channels that contribute to [Ca^2+^]_i_ signaling in response to CaSR stimulation

### CaSR activation upregulates the expression of TRPC3 and TPRC6

**Figure 5:**
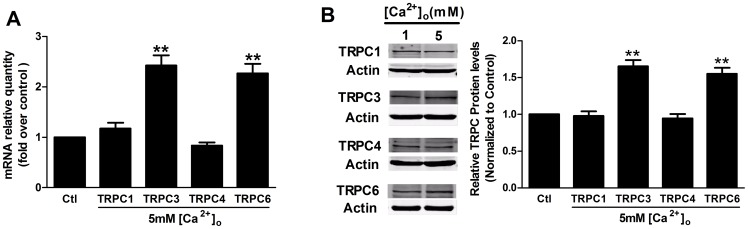
The influence of CaSR activation on TRPC mRNA levels and protein expression. Human MCs were starved for 24-free medium prior to stimulation with 1 mM (Ctl) or 5 mM [Ca^2+^]_o_ for 24 h. (A) Real-time PCR experiments showed that the administration of 5 mM [Ca^2+^]_o_ for 24 h significantly increased the TRPC3 and TRPC6 mRNA levels but did not affect the mRNA levels of TRPC1 or TRPC4 (**p<0.01 vs. Ctl, n = 3). (B) Representative Western blot and corresponding quantitative analysis showing that treatment with 5 mM [Ca^2+^]_o_ for 24 h increased the TRPC3 and TRPC6 protein expression but did not affect the protein expression of TRPC1 or TRPC4 (**p<0.01 vs. Ctl, n = 3). Asterisks indicate the statistical significance (**p<0.01), with respect to 1 mM [Ca^2+^]_o_ conditions (Ctl). Data are shown as the means ± SEMs.

Because CaSR activation has been shown to induce TPRC1 overexpression in MCF-7 cells [2] and TRPC3 overexpression in salivary gland cells [10], we examined the effects of CaSR activation induced by high [Ca^2+^]_o_ on the expression of TRPC mRNA and protein, including TRPC1, TRPC3, TRPC4 and TRPC6, which have been identified in human MCs [16]. Real-time PCR experiments showed that, in contrast with the control condition of 1 mM [Ca^2+^]_o_, treatment with 5 mM [Ca^2+^]_o_ for 24 h significantly increased the TRPC3 and TRPC6 mRNA levels by 142.10% and 126.77%, respectively (p<0.01; n = 3; Fig. 5A). Correspondingly, treatment with 5 mM [Ca^2+^]_o_ for 24 h significantly increased the TRPC3 and TRPC6 protein expression by 65.48% and 55.28%, respectively (p<0.01; n = 3; Fig. 5B). However, this treatment did not lead to increases in TRPC1 or TRPC4 expression (p>0.05; n = 3; Fig. 5).

### TRPC3 and TPRC6 are required for the CaSR agonist-induced [Ca^2+^]_i_ increase

**Figure 6:**
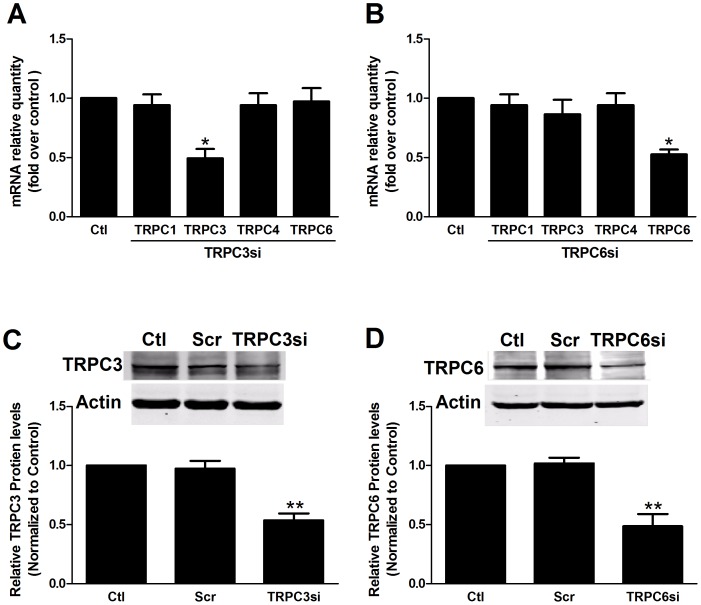
The specificity and efficiency of TRPC3 and TRPC6 knockdown. (A, B) Real-time PCR experiments showed that the TRPC3 siRNA (A) and TRPC6 siRNA (B) decreased the mRNA expression of TRPC3 and TRPC6, respectively (*p<0.05 vs. Ctl, n = 3), without affecting other TRPC channels (p>0.05, n = 3). (C, D) Western blot experiments showed that transfection with TRPC3 siRNA (C) and TRPC6 siRNA (D) reduced TRPC3 and TRPC6 protein expression (**p<0.01 vs. Scr, n = 3), respectively, without affecting other TRPC channels (p>0.05, n = 3) compared with transfection with scramble siRNA. Asterisks indicate the statistical significance (**p<0.01). Data are shown as the means ± SEMs.

**Figure 7:**
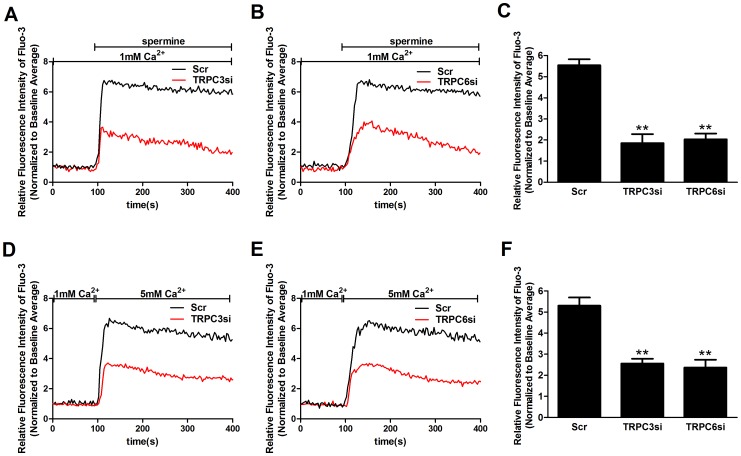
TRPC3 and TPRC6 are involved in the CaSR agonist-induced [Ca^2+^]_i_ increase. [Ca^2+^]_i_ dynamics were monitored by Fura-3 fluorescence methods. (A, B) Representative traces showing that transfection with TRPC3 siRNA (A) or TRPC6 siRNA (B) significantly inhibit the 3 mM spermine-induced [Ca^2+^]_i_ increase (p<0.05 vs. Scr, n = 5), respectively, in contrast with cells transfected with scrambled siRNA. (C) Summary of data showing that transfection with TRPC3 siRNA or TRPC6 siRNA significantly inhibits the average value of the plateau of the 3 mM spermine-induced [Ca^2+^]_i_ increase (**p<0.01 vs. Scr, n = 5), respectively. (D, E) Representative traces showing that transfection with TRPC3 siRNA (D) or TRPC6 siRNA (E) significantly inhibits the 5 mM [Ca^2+^]_o_-induced [Ca^2+^]_i_ increase (p<0.05 vs. Scr, n = 5), respectively, in contrast with cells transfected with scrambled siRNA. (F) Summary of data showing that transfection with TRPC3 siRNA or TRPC6 siRNA significantly inhibits the average value of the plateau of the 5 mM [Ca^2+^]_o_-induced [Ca^2+^]_i_ increase (**p<0.01 vs. Scr, n = 5), respectively. Data are shown as the means ± SEMs. The results were from at least three independent experiments, and each experiment measured 20 to 40 cells.

To investigate whether TRPC3 and TRPC6 are involved in [Ca^2+^]_i_ increase induced by CaSR activation, we used siRNA technology to downregulate TRPC3 and TRPC6 expression in human MCs. The specificity and efficiency of TRPC3-siRNA and TRPC6-siRNA was confirmed by real-time RT-PCR and Western blot analyses, indicating that this procedure decreased the expression level of endogenous TRPC3 and TRPC6 without affecting other TRPC channels (Fig. 6A–D). Compared with cells transfected with scrambled siRNA, transfection with TRPC3 siRNA and TRPC6 siRNA partially, but significantly, inhibited the spermine-induced [Ca^2+^]_i_ increase by 66.47% and 63.20%, respectively (p<0.05, n = 3; Fig. 7A–C). TRPC3 and TRPC6 knockdown also attenuated the [Ca^2+^]_o_-induced [Ca^2+^]_i_ increase (Fig. 7D–F). Transfection with scrambled siRNA did not alter [Ca^2+^]_o_- and the spermine-induced [Ca^2+^]_i_ increase compared with the non-transfected control (data not shown). Taken together, these results strongly suggest the requirement of TRPC3 and TRPC6 in the CaSR agonist-induced [Ca^2+^]_i_ increase.

### The release from calcium stores is not essential for TRPC3- and TRPC6-mediated calcium influx by CaSR activation

**Figure 8:**
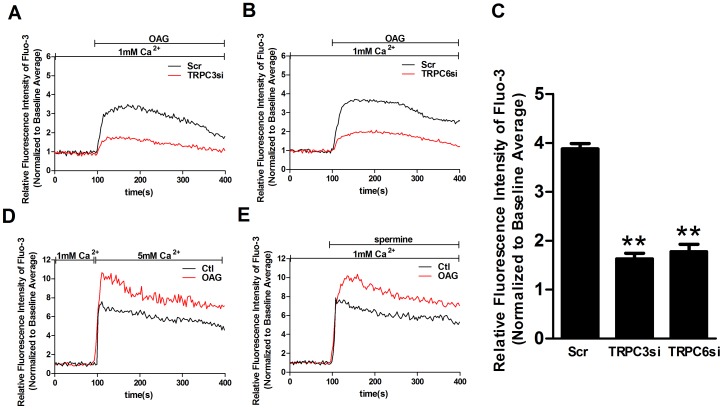
CaSR agonist-mediated Ca^2+^ entry is likely through receptor-operated Ca^2+^ channels in human MCs. [Ca^2+^]_i_ dynamics were monitored by Fura-3 fluorescence methods. Representative traces are shown in A-D. (A, B) Representative traces (A–B) and summary of data (C) showing that transfection with TRPC3 siRNA (A) or TRPC6 siRNA (B) significantly reduces the 100 µM OAG-induced Ca^2+^ influx (p<0.01 vs. Scr, n = 4), respectively, compared with transfection with scrambled siRNA. Data are shown as the means ± SEMs. (D, E) The 5 mM [Ca^2+^]_o_(D)- and spermine(E)-induced [Ca^2+^]_i_ increase is significantly enhanced by pretreatment with 100 µM OAG for 20 min (p<0.05 vs. Ctl, n = 4). The results were from at least three independent experiments, and each experiment measured 20 to 40 cells.

TRPC3 and TRPC6, as receptor-operated channels, can be activated by DAG and mediate ROCE in a variety of cell types. Therefore, we tested whether TRPC3 and TRPC6 can be activated by OAG, a membrane-permeable DAG analogue, in human MCs. As expected, the OAG-induced Ca^2+^ influx was significantly reduced by transfection with TRPC3 siRNA and TRPC6 siRNA compared with transfection with scrambled siRNA (Fig. 8A-C). Importantly, OAG significantly enhanced the [Ca^2+^]_o_- and spermine-induced [Ca^2+^]_i_ increases (Fig. 8D and 8E), suggesting that CaSR agonists likely evoke calcium entry via receptor-operated channels.

**Figure 9:**
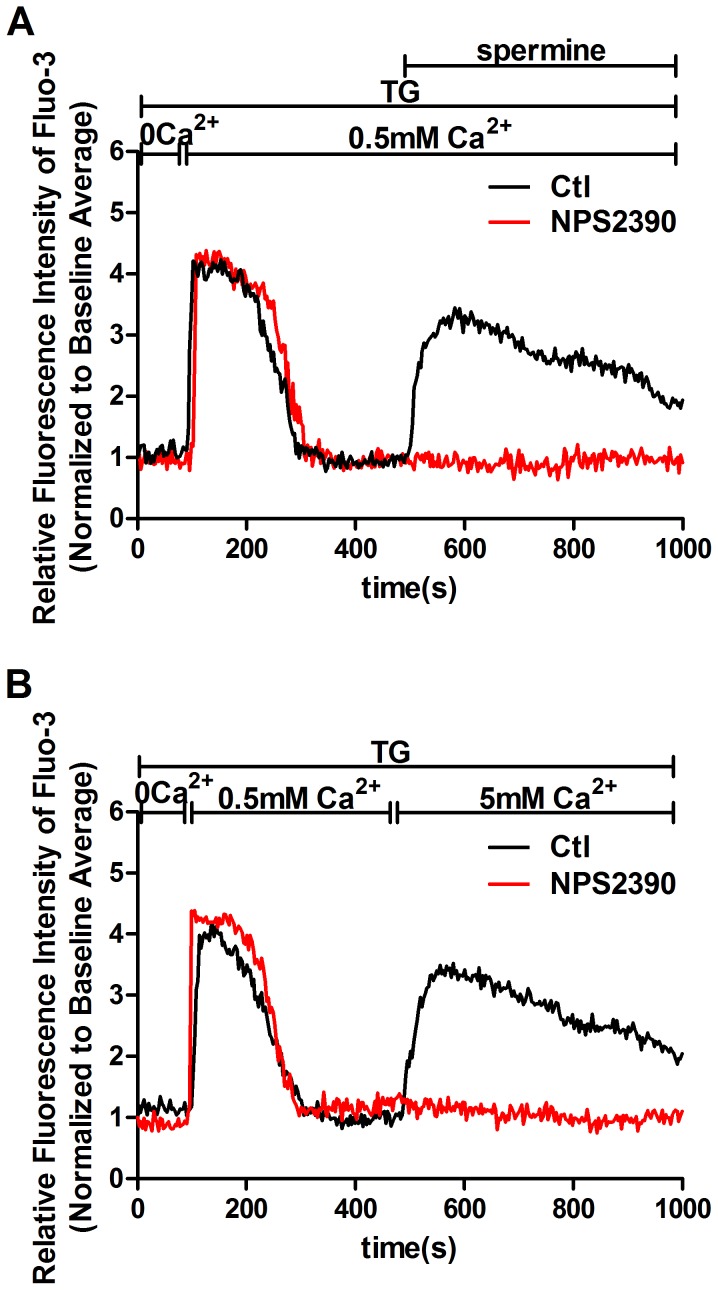
CaSR agonists mediate Ca^2+^ influx in store-independent manner. [Ca^2+^]_i_ dynamics were monitored by Fura-3 fluorescence methods. Representative traces are shown in A–B. (A) In the presence of 1 µM TG, a change in [Ca^2+^]_o_ from 0 to 0.5 mM induces a rise in [Ca^2+^]_i_, and then, addition of 3 mM spermine evokes an additional substantial increase in [Ca^2+^]_i_ under conditions where Ca^2+^ stores are already depleted. The store-independent calcium influx induced by 3 mM spermine was blocked by pretreatment with 10 µM NPS2390 (p<0.05 vs. Ctl, n = 5). (B) A restoration of [Ca^2+^]_o_ from 0 to 0.5 mM induces a rise in [Ca^2+^]_i_ in the presence of 1 µM TG, and then, a change in [Ca^2+^]_o_ from 0.5 to 5 mM evokes an additional substantial increase in [Ca^2+^]_i_ under conditions where Ca^2+^ stores are already depleted. The store-independent calcium influx due to 5 mM [Ca^2+^]_o_ was blocked by pretreatment with 10 µM NPS2390 (p<0.05 vs. Ctl, n = 5). The results were from at least three independent experiments, and each experiment measured 20 to 40 cells.

To further demonstrate that calcium influx stimulated by CaSR agonists does not require the release of calcium stores, we depleted stores with TG before CaSR stimulation. TG blocks Ca^2+^-ATPase located in the membrane of the endoplasmic reticulum (ER) and other intracellular vesicular store compartments. As shown in Fig. 9A, in the presence of 1 µM TG, a restoration of extracellular calcium from 0 to 0.5 mM induced an expected rise in [Ca^2+^]_i_, which was due to SOCE, and 3 mM spermine evoked an additional substantial increase in [Ca^2+^]_i_ under conditions where Ca^2+^ stores were already depleted. However, the additional substantial increase in [Ca^2+^]_i_ was blocked by NSP2390, a CaSR antagonist. Similar results were obtained with a 5 mM [Ca^2+^]_o_-induced calcium influx (Fig. 9B). These results suggest that TRPC3- and TRPC6-mediated calcium influx by CaSR activation does not require the release of calcium stores.

### TRPC3 and TRPC6 contribute to [Ca^2+^]_o_-mediated cell proliferation

**Figure 10:**
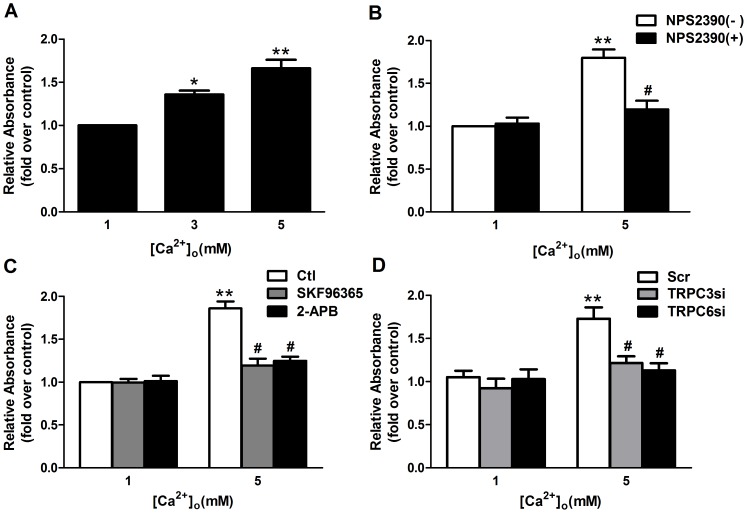
TRPC3 and TRPC6 are involved in high [Ca^2+^]_o_-mediated cell proliferation. Cell proliferation was measured by a Cell Proliferation ELISA BrdU kit. After starvation for 24-free medium, cells were incubated in the same medium supplemented with different [Ca^2+^]_o_ (1–5 mM) in the presence or absence of various inhibitors for 24 h. Untreated cells cultured in medium containing 1 mM Ca^2+^ were used as a control. (A) Incubation of cells for 24 h with 3 mM and 5 mM [Ca^2+^]_o_ increase proliferation of human MCs, respectively, compared with 1 mM [Ca^2+^]_o_ (Ctl) (*p<0.05, **p<0.01 vs. Ctl, n = 3). (B) Pretreatment with 10 µM NPS2390 significantly reduces the 5 mM [Ca^2+^]_o_-induced cell proliferation (**p<0.01 vs. Ctl, **#** p<0.05 vs. 5 mM [Ca^2+^]_o_ without NPS2390, n = 3). Incubation with NPS2390 alone do not affect cell proliferation at 1 mM [Ca^2+^]_o_ (p>0.05, n = 3). (C) The [Ca^2+^]_o_-mediated cell proliferation is significantly inhibited by pretreatment with 50 µM SKF96365 or 100 µM 2-APB (**p<0.01 vs. Ctl, #p<0.05 vs. 5 mM [Ca^2+^]_o_ without inhibitors, n = 3). (D) Transfection of TRPC3 siRNA and TRPC6 siRNA significantly attenuate the promotion of proliferation by 5 mM [Ca^2+^]_o_ (**p<0.01 vs. Scr+1 mM [Ca^2+^]_o_, #p<0.05 vs. Scr+5 mM [Ca^2+^]_o_), respectively, compared with scramble siRNA treated with 5 mM [Ca^2+^]_o_. Cells were transfected with TRPC3 siRNA, TRPC6 siRNA or scrambled siRNA followed by treatment with 5 mM [Ca^2+^]_o_ for 24 h. Untransfected cells cultured in medium containing 1 mM Ca^2+^ were used as a control. Data are shown as the means ± SEMs.

Because a previous study has reported that [Ca^2+^]_o_ mediates mouse MC proliferation via activation of CaSR, we investigated the role of TRPC3 and TRPC6 in [Ca^2+^]_o_-mediated cell proliferation. As shown in Fig. 10A, incubation of cells for 24 hours with 3 mM and 5 mM [Ca^2+^]_o_ significantly increased proliferation by 35.95% and 66.24%, respectively, compared with 1 mM [Ca^2+^]_o_ (p<0.05 and p<0.01, n = 3). The cell viability was not affected under our experimental conditions (data not shown). Consistent with previous reports, the promotion of cell proliferation by [Ca^2+^]_o_ appeared to act through CaSR stimulation because pretreatment of cells with 10 µM NPS2390 significantly inhibited [Ca^2+^]_o_-mediated cell proliferation (Fig. 10B). Incubation with NPS2390 alone did not affect cell proliferation at 1 mM [Ca^2+^]_o_. The [Ca^2+^]_o_-mediated cell proliferation was significantly inhibited by pretreatment with TRPC channel blockers, 50 µM SKF96365 and 100 µM 2-APB (Fig. 10C). Furthermore, transfection of TRPC3 siRNA and TRPC6 siRNA significantly attenuated the promotion of proliferation by [Ca^2+^]_o_, respectively, compared with scramble RNA (Fig. 10D). Taken together, these data indicate that TRPC3 and TRPC6 play a role in cell proliferation induced by CaSR stimulation.

## Discussion

CaSR has been demonstrated to be present in several tissues and cells unrelated to systemic calcium homeostasis, where it regulates a series of diverse cellular functions [1]. A previous study showed that CaSR is localized not only in the renal tubules but also in the glomeruli [20]. Further work indicated that CaSR is functionally expressed in mouse mesangial cells and modulates cell proliferation [8]. Unfortunately, no reports have been made on the mechanism by which CaSR induces calcium-dependent signaling in MCs. In this study, we demonstrate that CaSR is functionally expressed in a human MC cell line. Further, our data reveal that CaSR activation induces an increase in [Ca^2+^]_i_ via both calcium entry by TRPC3 and TRPC6 and release from intracellular stores. Furthermore, TRPC3 and TRPC6 are associated with CaSR activation-induced cell proliferation in human MCs.

CaSR can be activated by two types of agonists. Type I agonists are divalent cations, such as Ca^2+^ and Mg^2+^, which directly activate the receptor. As shown in this study, the EC_50_ of Ca^2+^ in human MCs is 4.93 mM, close to that of HEK-293 cells transfected with the human CaSR (4.1 mM) [21]. The type II agonists, such as spermine, amino acids, and ionic strength, are better referred to as modulatory substances, which allosterically increase the calcium affinity of the receptor [9]. The highly cooperative process of Ca^2+^ binding to CaSR allows CaSR to function as a sensitive detector of Ca^2+^, thereby quite easily distinguishing small (∼200 µM) fluctuations in the [Ca^2+^]_o_ [9]. Moreover, spermine is a uremia toxin that has been implicated as a potential mediator of chronic kidney disease-associated clinical abnormalities [22]. Therefore, CaSR could play an important role in MCs under physiological and pathophysiological conditions, although the typical physiological [Ca^2+^]_o_ is approximately 1.3 mM [23], much lower than that required CaSR activation in MCs as determined from our data.

Generally, exposure of cells to CaSR agonists commonly elicits a [Ca^2+^]_i_ increase through interactions between CaSR and PLC, which are mediated by the G_q/11_ subunits of heterotrimeric G proteins [9], [23]. CaSR activation may mobilize different Ca^2+^ sources in different cells. Upon CaSR simulation, intracellular Ca^2+^ is released from internal stores, as has been demonstrated in a variety of cell types; however, relatively little is known about the contribution of Ca^2+^ entry to the [Ca^2+^]_i_ increase. Here, we show that CaSR activation by agonists, both high [Ca^2+^]_o_ and spermine, produces a PLC-dependent [Ca^2+^]_i_ increase in human MCs. The CaSR activation-induced [Ca^2+^]_i_ increase is composed of an initial rapid increase and followed by a sustained increase, consistent with a previous study in mouse MCs [8]. The overshooting peaks may represent intracellular Ca^2+^ mobilization, and the sustained elevation may represent activation of Ca^2+^ influx. Indeed, our data indicate that CaSR stimulation induces TRPC-dependent calcium entry as well as calcium release from intracellular stores. Further, our results suggest that TRPC3 and TRPC6 may be responsible for the CaSR activation-induced calcium influx because of the following: (i) stimulation of CaSR by high [Ca^2+^]_o_ enhanced the expression of TRPC3 and TRPC6, rather than of TRPC1 and TRPC4, and (ii) siRNA targeting of TRPC3 and TRPC6 attenuated the CaSR activation-induced calcium influx. Previous patch clamp experiments revealed that stimulation of CaSR induces TRPC-like nonselective cation currents in HEK293 cells stably transfected with CaSR [24] and in MCF-7 breast cancer cells [25]. Moreover, several isoforms of TRPC channels, dependent on the cell type, have been implicated in the CaSR activation-induced Ca^2+^ entry, such as TRPC3 in salivary ductal cells [10], TRPC1 in MCF-7 breast cancer cells [2] and keratinocytes [12], and TRPC6 in aortic smooth muscle cells [11] and cardiac myocytes [4]. These studies support the contribution of TRPC channels in the CaSR activation-induced Ca^2+^ entry.

In MCs, as in other cell types, TRPC3 channels and TRPC6 channels are considered to be ROCs [16], [19], whereas TRPC1 channels and TRPC4 channels are SOCs [26]. Given that a functional hallmark of ROCs is that they can be directly activated by DAG without depleting intracellular stores, CaSR agonists may be able to induce the activation of ROCs because PLC-mediated DAG production following CaSR stimulation has been demonstrated in a number of studies [1], [9], [27], [28]. In this study, we show that OAG, a membrane-permeable DAG analogue, significantly enhances the CaSR agonist-induced [Ca^2+^]_i_ increase. Moreover, further experiments with TG to deplete intracellular stores before CaSR stimulation revealed that the effects of CaSR agonists on [Ca^2+^]_i_ still occurred, suggesting that calcium influx stimulated by CaSR agonists do not require depletion of intracellular stores. This idea is supported by the results obtained in osteoclasts and aortic smooth muscle cells, where CaSR activation mediates ROCE [11], [14]. Furthermore, our findings is concordant with several observations that have shown that TRPC3-TRPC6 channels mediate store-independent Ca^2+^ entry in prostate smooth muscle cells [29], MDCK [30], and cardiac myocytes [31]. However, in other cell types, such as MCF-7 breast cancer cells and keratinocytes, CaSR activation mediates SOCE [2], [3], suggesting that CaSR stimulation mediates Ca^2+^ entry in a cell-specific manner. In this study, we cannot exclude the possibility that SOCE occurs simultaneously with ROCE upon CaSR activation because CaSR stimulation induces Ca^2+^ release from intracellular stores and can thereby directly or indirectly affect the TRPC3 and TRPC6 activities [19].

Finally, we determined the role of TRPC3 channels and TRPC6 channels in CaSR stimulation-induced cell proliferation, showing that both pharmacological blockage and siRNA knock down of TRPC3 and TRPC6 inhibited high [Ca^2+^]_o_-induced mesangial cell proliferation. A similar role was described in MCF-7 breast cancer cells. However, whether the role of TRPC3 and TRPC6 in the CaSR stimulation-induced cell proliferation is mediated by changing intracellular calcium or results from multiple postreceptor responses has yet to be determined. Regardless, our study demonstrates that CaSR modulates extracellular calcium entry and proliferation via TRPC3/6 channels in human MCs.
